# Collectanea Medica: Consisting of Anecdotes, Facts, Extracts, Illustrations, &c.

**Published:** 1821-01

**Authors:** 


### COLLECTANEA MEDICA:

CONSISTING OF

### ANECDOTES, FACTS, EXTRACTS, ILLUSTRATIONS, &c.

*Relating to the History or the Art of Medicine, and the Auxiliary Sciences.*

---

Floriferis ut apes in saltibus omnia libant,

Omnia nos itidem depascimur aurea dicta.

---

### *Extracts of the Report from the Select Committee of the House of Commons on the Doctrine of the Contagion of the Plague, in 1819.*

*Veneris*, 19° *die Martij*, 1819.

Sir John Jackson, Baronet, in the Chair.

Dr. William Gladstone called in; and examined.

YOU are a practitioner in medicine?—Surgeon to the Naval Asylum at Greenwich.

Are you acquainted with the disease termed Plague in the Levant, and the contagion ascribed to it; and what is your opinion thereof? I was at Constantinople in 1806 and 1807; and, from having been then surgeon of his Majesty's ship the Endymion, I there saw some diseases of the plague, and a great variety of Asiatic fevers, highly infectious.

Was the plague at the time raging?—No: there is at all times more or less plague there.

At what time of the year?—It was in December, January, February, and March, prior to the forcing of the Dardanelles.

Will you describe to the Committee the characteristics of the plague?—It is a disease attended by sudden prostration of strength and spirits, great apprehension, and many symptoms of a malignant type. From the little apprehension the Greek physicians I saw have to come in contact with the plague, I was led to feel the pulse of their patients, which I did with considerable apprehension. I do not consider the plague more contagious from contact than through the medium of the atmosphere.

What was the consequence?—I felt no bad consequences.

How many plague patients do you suppose you felt the pulses of?—I saw three, and took opportunities of visiting them several times.

How was their pulse?—Very quick and full, frequently alternating, indicating great arterial disturbance. They were stout men; slaves belonging to the arsenal.

Had they buboes or carbuncles on their bodies?—One had an enlargement in the axilla.

A bubo?—Yes: and two of them died of the plague, I was afterwards informed.

From what you have seen of the plague with your own eyes, do you consider it as contagious?—I consider it as highly infectious. My opinion is, that it is equally so through the medium of the diseased atmosphere of a sick chamber, as by simple contact, by feeling the pulse.

What do you suppose is the cause of the plague at Constantinople?—Diseased constitution of the atmosphere, and other peculiar causes, such as effluvia and soil, which produce endemic diseases all over the globe; from the same causes as we have epidemic diseases in England; and from the circumstance of that city standing upon hills. Many of the houses are built on ground sloping to the south-west, consequently liable to the whole action of the south-west sun; all are badly ventilated. The streets are very narrow; and they do not, I believe, possess that grand source of health, common sewers.

Are you acquainted with the suburb of Pera?—Yes.

That suburb is chiefly inhabited by Europeans?—Chiefly by Europeans.

Is it true that there is generally less plague in that district than in any other?—Perfectly true.

To what do you ascribe that?—To the houses not being so close, or the streets so narrow.

Is there increased cleanliness in that district?—More, I think, in Pera than Constantinople.

What do you mean by infection?—Disease produced by a contagious state of the atmosphere. An epidemic state of the atmosphere produced by various causes: effluvia, virous effluvia, or by contact with a particular virus.

Have you ever heard of the plague being in Great Britain or Ireland?—Yes; both, during the disease of 1665, in Charles II.'s time. There is an order in council by the honourable privy-council, present the Lord Chamberlain, the Earl of Bath, Mr. Treasurer, Mr. Vice-Chamberlain, and Mr. Secretary Morris, ordering the College of Physicians to suggest measures, and give directions to prevent the spreading the infection of the plague.

Do you consider that was the real Levant plague?—No.

Why?—At that period, as far as I have been able to trace from a variety of old authors, there was scarcely such a thing as a common sewer. The privies were accumulated under every house, probably not emptied for years; and an order was given to empty them once a-month. That order originated, I believe, in the College of Physicians, after the spreading of the plague.

As you do not consider that to have been what is properly termed the Levant plague, to what do you ascribe the sickness in 1665?—To the narrowness of the streets, accumulation of filth, and want of ventilation; and, probably, a diseased constitution of the atmosphere at that period.

Then you consider it was not the Levant plague; that it was not imported, but that it originated in England?—Yes; I believe that it originated in England.

Do you found your idea of its not being the Levant plague from any historical description or nosological character of the disease?—I have not been able to trace any decided fact of its importation: a circumstance of so much importance and general interest, if true, would, I think, have been marked by every medical writer and historian of the time. I look upon the narrowness of the streets, the filth of the city, the want of common sewers, and the state of the atmosphere at that period, to have been the causes; but particularly the want of common sewers.

Was it attended with buboes and carbuncles?—Yes, as far as I am able to learn; in some cases, not generally.

Did you ever see cases of typhus attended with buboes?—Yes, in the first instance, in some inflammatory constitutions.

Frequently?—Not frequently.

Have you seen the carbuncles in typhus?—I have seen an enlargement in the groin: the carbuncle, in general, comes on the back between the shoulders.

Have you ever heard of the plague in Great Britain and Ireland since 1665?—There was a disease in Dublin something similar, but I have not any correct information as to it.

In what year?—I do not know exactly; I believe soon after the plague in London, but I am not sure.

Do you consider that our quarantine establishments have kept the plague from being introduced into Great Britain or Ireland?—No, I do not; I cannot say they have. From having been frequently under quarantine restraint myself, I have made it my business to visit most of the lazarettos between Gibraltar and Constantinople; but the source of disease is more frequently seen among the Greek vessels that carry cargoes to Marseilles, of which there was a great many sailed soon after the blockade by the British squadron under Sir John Duckworth.

If plague-infection was introduced into bales of goods in the Mediterranean, do you suppose that the present conveniences and opportunities at quarantine establishments in Great Britain sufficient to purify them from the infection?—I consider the lazarettos, as far as I am able to learn, particularly inefficient in fitment for that purpose; I mean with respect to ventilation and ballast.

Do you consider that the Levant plague can exist in a British atmosphere?—I think that is very doubtful; but I think there is great encouragement to nurse disease, if any is imported into the lazarettos. There are some of the lazaretto ships, as I am given to understand, where the shingle-ballast has not been shifted for many years; and in many instances fevers have been produced and nursed from this case, even in our men-of-war. The men-of-war formerly used to be ballasted with shingles: on turning this ballast, it has produced fever in several of the ships; that I have seen myself; but it is well recorded.

What kind of fever?—The usual fever of the station they happened to be on, or the place they were at: not the plague. It is a well-known fact, established among maritime people, that the health of our fleets, our troops in transports, and seamen in merchant-vessels, have all risen in proportion to the degree of perfection at which we have arrived in cleanliness and ventilation.

Have you, in your enquiries, ever heard of a plague case having arrived at, or been seen, in any lazaretto in Great Britain?—Never.

Do you not suppose, that if the infection of plague had been imported in any bales of goods from the Mediterranean, which were opened for the purpose of being ventilated at lazarettos, that if the plague had existed therein, and was contagious, those expurgators would become themselves infected with the plague?—Certainly.

Would the circumstance of plague not having been seen for so many years in Great Britain, or in lazarettos, give sufficient confidence for concluding that the plague cannot exist in a British atmosphere?—I should think it would not give sufficient confidence; it would create great alarm, if known to exist.

You still suppose it might exist?—I think that is a doubtful point.

Is not plague in plague-countries periodical in its beginning and end?—Yes.

What is the state of atmosphere that you conceive compatible and not compatible with the existence of plague?—I look upon it, that in cold dry weather the plague does not so frequently exist. In hot weather, after floods, when the rivers, such as the Nile, have overflowed, and left marshes and ponds, the action of the sun in summer on such marshes and moist ground always produces disease, and frequently in the Levant plague.

What sort of temperature do you consider necessary to the existence of plague?—In the cases of plague I saw at Constantinople, the thermometer stood about the freezing point, from 26 to 30: it was in the winter.

Was the plague prevalent at that state of temperature?—No, it was not; but there were always some cases there.

There were cases even during that state of the year?—There are always some cases at Constantinople.

Was you at Constantinople, or any other part of the Levant, at any period of the year when the plague was raging violently?—No.

Do you happen to know what the state of the atmosphere is, in which it is proposed to act most violently?—I believe a high temperature, from 66 to 76 and upwards.

Is there any reason, when there is a hot moist air in this country, and the temperature rises to that point, that the plague should not exist in this country as well as in any other?—I do not think the summer-heat ever rises so high in England as in those latitudes.

As it appears that it existed at Constantinople when the temperature was nearly at the freezing-point, is there any reason why it should not exist in the same temperature in any other country?—In most other countries, and particularly in England, the houses are better ventilated, more cleanly, and well drained.

Then you mean to state, that the probability of its existing in England depends on ventilation, and the cleanliness of the people of the country?—Not entirely, but in a great measure.

Do you consider the plague in its character as infectious or contagious?—I consider it equally infectious through the medium of the atmosphere of a sick chamber as from simple contact, having experienced it so far as having felt the arm of a patient under plague.

As you appear to consider the plague as connected with the state of the atmosphere, when the atmosphere is in that state that it admits of the existence of plague, do you then consider it an infectious disorder: I mean, when the air is in that state as to produce a liability to infection by the touch?—I think it is impossible to come in contact with a plague-patient, without inhaling the atmosphere of his chamber; and that we are more susceptible of infection by the membranes of respiration than those of the fingers.

Do you confine it to the sick chamber, or to the general atmosphere?—The surrounding atmosphere of the chamber: the atmosphere of a sick chamber is more infectious than any other.

From any experience that you have had of the plague, have you seen instances of its being communicated any other way than by the touch?—Not in any way; I have never seen an instance of its being communicated.

You have stated, that you were present when there were cases of plague?—When there were plague-patients at Constantinople, I made interest to view them with the Arabian physicians.

State the circumstances of your visit?—I saw him approach the patient, and feel the pulse, without the least fear; and, upon the second view, but with some apprehension of contagion, I was induced to do the same.

In what stage of the disease?—One patient had been ill a week, and was recovering; the others had been ill two or three days; I understood they died: the one of the week's illness I understood to have recovered.

Were buboes and carbuncles on these?—One in the axilla.

Were these all the cases that came under your observation?—These were all the cases that came under my eye that were decidedly plague: I saw a great variety of Asiatic fevers.

Do you attribute its not being communicable by contact, to the state of the air?—I consider that the plague is not more liable to be communicated by contact, than by inhaling the atmosphere in a sick room.

Then you believe it is equally communicable by both, either by contact or inhaling?—It is impossible to approach to contact without inhaling the atmosphere of a sick chamber.

What length of time was you at Constantinople?—I was there three or four months.

Do you consider these points of metaphysical temperature of air so useful and decisive in the investigation of disease, as the plain fact whether the disease has ever existed or not for many years?—No, I do not.

Then you consider the plain fact where it has not existed, the best criterion?—The best criterion; but I do not consider we have arrived at sufficient knowledge with respect to air, so as to ascertain these facts perfectly.

As you touched the plague-patients and felt their pulses, and did not receive the disorder, would not that rather convey the idea that the plague was not contagious?—That would depend upon the state of the atmosphere, and on the state and constitution of the individual who had touched the patient; and whether there was a predisposition to receive the disease or resist it.

Did you make the subject of the plague your study while you was in these places?—From being frequently under the restraint of quarantine, I thought a good deal upon the subject, and read a good deal.

Did you pursue that by inquiries in the country?—By inquiries at the places we touched at. I made a point of visiting the lazaretto when I was on quarantine, and permitted to do so: where there was no lazarettos, I made a point of getting information from medical practitioners.

Were these practitioners Greeks or Turks?—In Constantinople there were Greeks, Armenians, and Arabians; some few Italians at Pera.

Were they people who entertained the same opinion on the subject of predestination as the Turks?—Most had not; some had; that is an opinion they do not avow.

I would ask whether they stated to you that they considered there was any danger in the use of the clothes of the persons who had died of the plague?—Yes; certainly.

Then you infer, that the plague may be conveyed by clothes, or other things than the touch?—Certainly.

Have you any means of knowing what length of time packages may be conveyed, supposing the air not to operate upon them, or any clothes or materials used by persons having the plague, before they lose the power of communicating the infection: state, either from your own experience or information?—That entirely depends on the ventilation of the packages.

If the packages are not subject to ventilation, you are of opinion that the plague may be conveyed?—It depends on the state of the atmosphere. What I conceive as to cotton is, that it depends on the state of the cotton when it is brought on-board. The Turks are a people very superstitious: when a person dies of the plague, they put the corpse into a shell, and sometimes the buboes discharge matter on carrying a corpse to the grave; this matter comes in contact with the dust: or, if that dust should afterwards be mixed with cotton, and that cotton comes to England and meets with a diseased atmosphere, I cannot answer for the long conveyance, without ventilation, destroying the infection.

You have no reason to conclude it could not?—I have no reason to conclude it could not. I think a lazaretto, properly fitted-up with ventilating apparatus, so as to cause a current of air to be constantly percolating through the cargoes, the vitality of any contagion that might be conveyed to England must be soon destroyed by that means.

Are not animal substances, such as goats' skins, particularly susceptible of infection?—They are; goat-skins and hare-skins, and Turkey carpets.

Do you think that the bales of goods which are closely packed and come from Constantinople, admit of such a ventilation, without being completely opened, as to give security?—No; it depends much on the state of ventilation at the lazarettos. I am of opinion, that the airing process might be as efficiently performed as it now is, in a much shorter period, by a different fitment, attending to the state of the ballast and hold, which in every ship is important, but in lazarettos most particularly so.

You stated, that you thought that one of the things that contributed to the plague was the stagnant water?—It always contributes to disease.

Do you know that the plague is frequently prevalent in Egypt?—Very well.

Do you happen to know at what period the Nile rises and falls?—I believe it begins to fall about August. I have not a perfect recollection of the history of the Nile.

Do you not know that the Nile rises during the summer in hot weather, and subsides during the winter?—It subsides during the autumn, I think.

Is plague prevalent in Egypt during the winter and autumn?—It is more prevalent in summer than in winter.

That is, it is prevalent in Egypt at a time when stagnant water is not found?—Yes, at all times.

After what you have stated, I should like to have your opinion, as a physician, whether you would take on yourself to advise, on your own responsibility, the relaxation, or the material relaxation, of any of the precautions in this country to prevent the communication of the plague?—That is a serious question. Though I have not a doubt but what the quarantine might be with safety considerably diminished, under certain regulations of ventilation and fitment, in lazarettos; yet it is a thing of so much importance, that it ought to be proceeded in with the greatest caution.

Do you think any distinction might be taken between persons and goods in quarantine?—Yes, I do.

In what period do you think persons would be safe from Smyrna?—As much quarantine in general as the men-of-war perform; three days. At Malta, coming from the Morea, I remember bringing a passenger who was very anxious to go on-shore, and they let him out in three days.

Did you ever hear of inoculation for the plague?—Yes, I have, by Dr. White, whom I knew well: the patient died under the disease. I am not sure it was the plague, or whether the disease he died of was from inoculation.

Do you make a distinction between ships of war and merchant ships? —Certainly.

Do you consider it more likely that the infection should be received in the internal part of a bale, or the outside?—That depends on the state of the bale of cotton; the state it was embarked in, whether moist or perfectly dry.

Is it not likely to exist in the internal part of a bale?—Certainly, infection is.

And therefore, if it does so exist, according to your doctrine, would it not communicate plague, unless properly purified and ventilated?—That I consider very doubtful; but I should be very desirous to avoid the risk, by having it ventilated.

Do you believe that the plague can be imported into Great Britain?—I should think it possible, certainly.

Do you think it likely?—No; and I think the vitality of the disease might be soon destroyed, if it was.

And therefore you consider it could not long exist in England?—I think it could not long exist in England.

If the air was in a state which admitted of its existing at all, and it was once communicated, what reason is there to believe it would not be generally communicated: why would it not spread?—The disease would be weakened, in the first instance, in the lazarettos, by ventilation and fumigation.

I am assuming a case where there is no lazaretto?—I see no reason why it should not spread, except that the English people are more cleanly, better ventilated in their apartments; and the common sewers and drains carry off all filth, which is a great cause of the spreading of the plague in other countries.

If it got among the lower orders of people, who are extremely crowded together in large towns, might it not then?—Certainly; but they would be immediately separated.

Do you think the plague more infectious than the small-pox, or measles, or typhus?—Yes, perhaps more infectious than any other disease: I have never seen any infection from it; I have from all the other diseases named. In the hospital I have now charge of, I had, four yours ago, about eighty-two cases of measles of a mild character. I was desirous of two girls being infected; I placed them in the wards; neither of them took it. I changed their linen; they both took it, and passed the disease mildly.

Did you ever know cases of typhus in which there were buboes as in the plague?—In high inflammatory constitutions, I have seen an enlargement of the groin, probably a sympathetic enlargement, in the first stage of the disease.

Do you consider the buboes a distinctive character of the fever called the plague?—I should think so.

Is the typhous fever, such as we have it in England, a common disorder in those countries where plague prevails?—It is not so prevalent, but it is more fatal; the bilious remittent fever is more common.

The typhus exists distinctly from the plague, though not so common?—Distinctly, though not common.

Do you think that different regions, according to their different circumstances of climate, soil, and construction of cities, produce among the people, at certain seasons of the year, fevers of a particular character?—Yes, certainly.

Do you think that one case of such fever being contagious, and the contagion transported to another country, where the circumstances are different, they would establish themselves in the other country?—No; only partially, if at all.

Is that your general opinion as to the probability of the plague being transported from the Levant, and communicated to other countries?—Yes; although, if it was imported into England during a diseased condition of the atmosphere, it might do great mischief before the vitality of the disease was overcome.

But you think it could not long survive its importation?—Not long.

And so you think of all other fevers?—All others that are not endemic of the country.

All other fevers characterized by particular circumstances?—Yes.

Do you suppose the atmosphere of England has been applicable to the receiving or generation of plague for the last one hundred years?—No; this country, and every part of the world inhabited, has been more cultivated, underwood near cities has been cleared away, and swamps drained, which has contributed much to rendering the disease milder.

Does not plague occur near the Mediterranean, where quarantine-laws are rigid?—Yes.

Do you suppose the plague to be introduced into these places, or generated in the places themselves?—I have never been able to ascertain as a fact the introduction of plague into any place: I have heard it attributed to men and to goods, but never of my own knowledge.

Have you ever heard that the plague which prevails so generally upon the coast of the Levant, has been spread eastward over the continent of Asia?—I believe it has made some progress, but not always eastward.

I should be glad to know your opinion, founded on your general knowledge, why the plague should not proceed eastward through the continent of Asia by land, as probably it is communicated westward, by persons and goods transported on-board a ship?—I consider that as entirely depending upon a diseased state of the atmosphere in these places; and, as to its being communicated westward by persons and goods, I never knew it so communicated.

You must have heard that the plague is frequent in Aleppo, and that the caravans proceed regularly with goods in bales from Aleppo eastward through the continent of Asia: have you ever heard of the plague being communicated by these caravans to the eastern country?—Never.

Why should not the plague be carried in bales of goods transported to Asia eastward, as well as brought by goods or persons on-board ship westward?—I see no reason why it should not.

Does your knowledge of the practice of countries eastward enable you to say whether the transportation of goods by caravans is calculated in any way to prevent the communication of the plague?—No: but I have seen the unloading of a caravan; the goods are not so closely packed in caravans as in Levant ships. You are aware of the mode they adopt in ships: the cargoes are screwed down; they often raise the beams of a ship in forcing the goods down; and consequently they are more liable, from their close stowage, to retain infection, if infection is embarked.

But the plague might as well be acquired by a caravan as brought by a ship?—Certainly.

You say you was present at the unpacking of the caravans?—Yes.

Where?—At Constantinople.

From whence did they come?—I have seen one, I believe from Aleppo. I was only present at the unpacking of one, and I am not aware where it came from: they pick up goods, the same as our waggons, every where on the road, I was told.

Do you know that they come through infected countries?—I am not aware of that.

Are you not surprised that the expurgators of goods in lazarettos in Great Britain have never received the plague?—I should conclude, from its not having been imported.

Then you suppose it has not been imported since the establishment of lazarettos?—Yes, I suppose so.

Do you think the epidemic disease under certain circumstances may become contagious?—Yes, it certainly may become infectious.

Do you suppose that the plague has been imported prior to the establishment of lazarettos in Great Britain?—I never heard that it has been ascertained as a fact.

If it had been imported, would it naturally have occasioned the plague in the community?—Not naturally: it might have been destroyed by the climate, or by management.

How long do you consider the infection of the plague may be latent in bale goods?—I cannot exactly state what length of time it may remain latent in bale goods.

Do you know that there is no ascertained account of the introduction of the plague by importation, previous to the quarantine-laws?—Not any that I am acquainted with.

Do you think that the concurrent testimony of all the old historians was merely vague report?—I am not competent to decide; I cannot speak as to that question.

Would you not rather be governed by modern facts than historical reports?—Certainly I would.

Do you consider the typhous fever a species of the plague?—Not exactly, but partaking of a highly malignant fever.

Does not Cullen call the plague a high state of typhous fever?—Yes, a high state of contagious typhus; but he never saw it.

